# Autonomic Responses During Acute Anterior Versus Inferior Myocardial Infarction: A Systematic Review and Meta-Analysis

**DOI:** 10.7759/cureus.48893

**Published:** 2023-11-16

**Authors:** Vaios Schismenos, Alexander A Tzanis, Georgios E Papadopoulos, Dimitrios Nikas, Ioanna Koniari, Theofilos M Kolettis

**Affiliations:** 1 Cardiology, University of Ioannina, Ioannina, GRC; 2 Internal Medicine, Metaxa Memorial Cancer Hospital, Piraeus, GRC; 3 1st Department of Cardiology, University Hospital of Ioannina, Ioannina, GRC; 4 Electrophysiology and Device Department, University Hospital of South Manchester NHS Foundation Trust, Manchester, GBR; 5 Cardiology, Cardiovascular Research Institute, Ioannina, GRC

**Keywords:** vagal response, sympathetic response, inferior wall myocardial infarction, acute anterior myocardial infarction, acute st-elevation myocardial infarction, meta-analysis, heart rate variability, autonomic responses

## Abstract

Autonomic responses elicited by myocardial infarction vary depending on the site of injury, but accurate assessment using heart rate variability during the acute phase is limited. We systematically searched PubMed without language restrictions throughout July 2023. We reviewed studies reporting autonomic indices separately for anterior and inferior infarcts, followed by a meta-analysis of those reporting the standard deviation of the inter-beat interval between normal sinus beats during the initial 24 hours after the onset of symptoms. Six studies were included, comprising 341 patients (165 anterior, 176 inferior infarcts), all with satisfactory scores on the Newcastle-Ottawa quality scale. The estimated average of the standardized mean difference (based on the random-effects model) was -0.722 (95% confidence intervals: -0.943 to -0.501), which differed from zero (z=-6.416, p<0.0001). This finding indicates sympathetic and vagal dominance during acute anterior and inferior infarcts, respectively, with excessive responses likely contributing to early arrhythmogenesis. Despite the amelioration of autonomic dysfunction by revascularization, infarct location should be considered when commencing β-adrenergic receptor blockade, especially after delayed procedures.

## Introduction and background

Acute myocardial infarction (MI) is often complicated by sustained ventricular tachyarrhythmias, which can have a lethal outcome [1]. Prompt revascularization has increased survival rates, but the acute phase of MI remains critical and constitutes a highly arrhythmogenic timeframe [2]. Although ischemia-induced ventricular tachyarrhythmias occurring prior to medical attendance carry an ominous prognosis, morbidity and mortality are also affected in hospitalized patients. Clinical data, derived from a cohort encompassing over 9000 patients, showed increased in-hospital mortality after sustained ventricular tachyarrhythmias recorded during the initial 24 hours post-MI [3]. Moreover, recent evidence indicates that long-term prognosis is also affected in survivors after hospital discharge [4]. Such a high impact of arrhythmogenesis during acute MI dictates the investigation of the underlying mechanisms, aiming at formulating strategies that will improve the overall prognosis.

The autonomic nervous system plays an important role in modulating cardiac electrophysiology during myocardial ischemia. Functional data derived after coronary artery occlusion in anesthetized cats [5] and dogs [6] demonstrated varying autonomic reflexes, depending on the location of ischemia. Such differences have been attributed to the uneven distribution of ventricular afferent nerve endings, as described in cats [7] and guinea pigs [8]. These findings were supported by clinical observations [9], demonstrating marked variation in autonomic responses accompanying acute MI. Although vagal activity opposes the detrimental effects of sympathetic inputs in this setting [10], bradycardia secondary to excessive vagal activation can also trigger ventricular tachyarrhythmias [11]. In early work [12], heart rate and blood pressure measurements in mobile coronary care units revealed autonomic disturbances in over 90% of patients; these consisted of vagal or sympathetic dominance, depending on the site of injury, both contributing to arrhythmogenesis [12]. This seminal work subsequently paved the way for research efforts that examined autonomic responses accompanying acute MI [13].

Heart rate and blood pressure changes during acute MI provide only an estimate of autonomic balance, whereas analysis of heart rate variability (HRV) yields a more accurate assessment of autonomic responses. This view is perhaps best exemplified in a cohort of 95 patients [14], in whom time-domain analysis of HRV indicated prominent vagal activation in inferior MI, despite comparable mean heart rate to anterior MI. However, observational studies reporting HRV early post-MI are scarce, some including a low number of patients, raising uncertainty about the statistical power to detect clinically meaningful differences.

Against this background, the present work examined the available data, aiming to provide further insights into the pathophysiology of acute MI. The relation between autonomic responses and infarct location also carries therapeutic implications regarding the long-debated topic of optimal timing for β-blockade initiation [15,16]. Toward these aims, we systematically reviewed studies reporting indices of sympathetic and vagal activity, separately for acute anterior and inferior MI. Studies reporting the standard deviation of the interbeat interval between normal sinus beats (SDNN) were entered into a meta-analysis; we selected SDNN, based on the straightforward and widely accepted methodology of calculating this index, facilitating the comparison across studies. Additionally, we reviewed reports providing other HRV indices in relation to the site of infarction, before and after revascularization.

## Review

We searched PubMed (National Institute of Health, USA) for relevant studies published throughout July 2023. The keywords used in our search strategy were "autonomic nervous system," "autonomic activation," "heart rate variability," "acute myocardial infarction," "acute coronary syndromes," "myocardial ischemia," "infarct location," "infarct site," "anterior," and "inferior."

Inclusion and exclusion criteria

Clinical observational studies were included if they examined adult patients diagnosed with acute MI, with HRV analyzed from Holter recordings. The timeframe for reporting was defined as the initial 24 hours after the onset of symptoms, with SDNN reported separately for anterior and inferior MI. No restrictions were set regarding publication date or language. The included studies were evaluated using the Newcastle-Ottawa quality scale, a collaborative project between the University of Newcastle, Australia, and the University of Ottawa, Canada, which has been thoroughly tested [17]. In this tool, each study is evaluated based on the patient selection process, the comparability between groups, and the ascertainment of the outcome. A score is given on eight items, facilitating quick visual assessment. 

Study design characteristics, clinical information, and outcome data were collected; data extraction and evaluation were conducted independently by two authors (V.S. and A.A.T.) and differences were adjudicated by consensus among all.

Meta-analysis

Heterogeneity among studies was assessed using the restricted maximum-likelihood estimator, along with the Q-test. Aided by the Jamovi software [18], studentized residuals and Cook's distances were applied as measures of outliers and overly influential studies, respectively. Those with residuals larger than the 100[0.95/2(number of studies)]^th^ percentile of a standard normal distribution were considered outliers, whereas those with Cook's distances larger than the median+6[interquartile range] were considered overly influential. The analysis was performed using the standardized mean difference (i.e., the observed difference divided by the standard deviation) as the outcome measure, with a random-effects model fitted to the data. The corresponding mean differences (with 95% confidence intervals) were calculated, followed by the construction of forest and funnel plots, whereas asymmetry of the latter was assessed using the rank-correlation and regression tests. Data analysis and reporting strictly adhered to the guides issued by PRISMA [19].

Studies included

**Figure 1:**
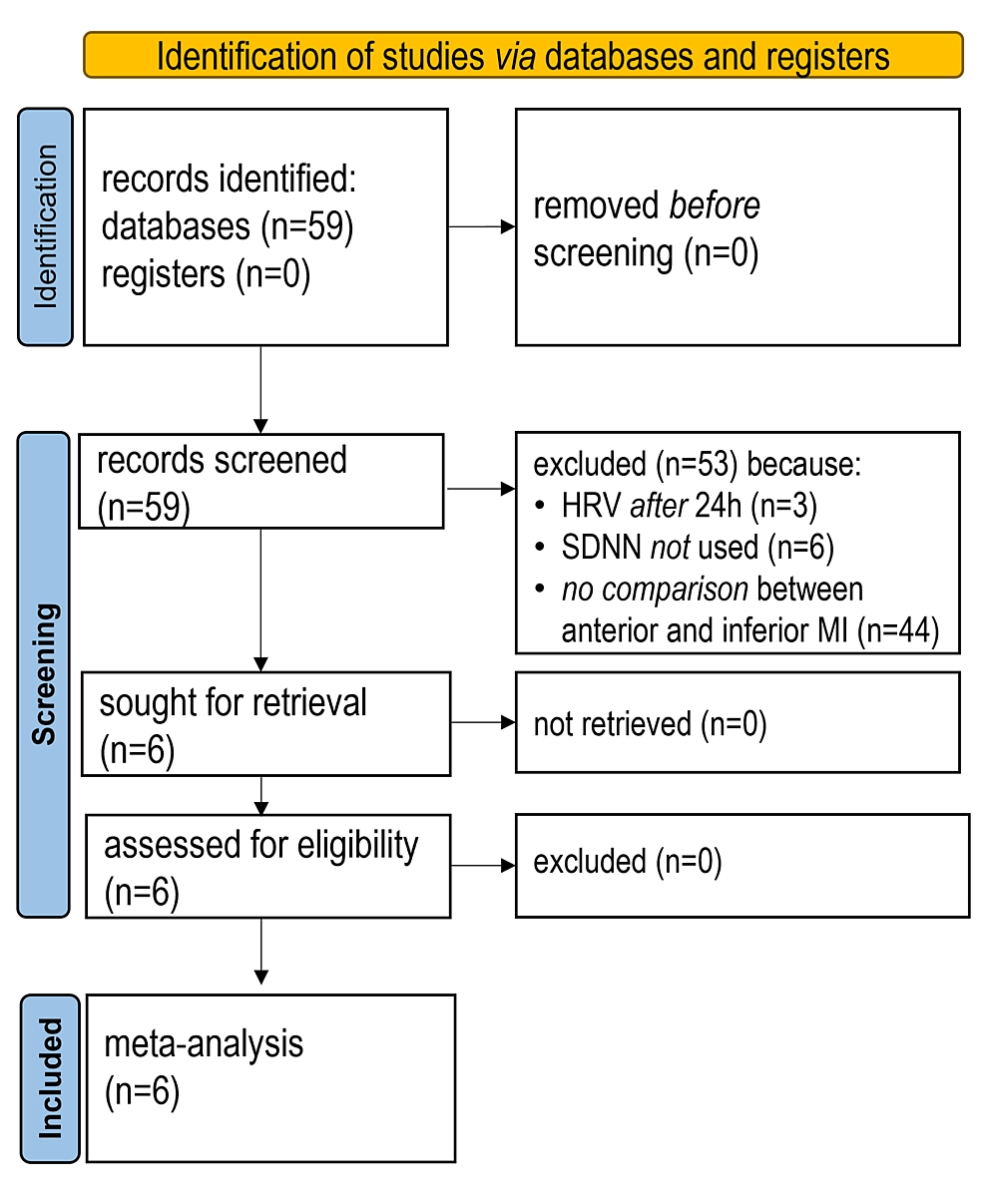
PRISMA flow diagram

After initial screening, the application of inclusion and exclusion criteria identified six eligible studies [20-25], reporting SDNN from long-term recordings (commenced on admission), as seen in the PRISMA flow diagram (Figure 1).

We also found four relevant studies, of which two [14,26] reported the number of times when successive heartbeat intervals exceeded 50 ms (pNN50) in the time-domain analysis, and two [27,28] reported the power of low- and high-frequency spectra. In both latter studies [27,28], revascularization was achieved by thrombolysis, whereas primary percutaneous coronary interventions (PCI) were used in two additional studies [29,30]. Their findings are discussed below, but a meta-analysis was not feasible, due to the small number of studies reporting each variable.

The studies reporting SDNN according to infarct location were entered in the meta-analysis, namely: (a) the study by Pipilis et al. [20] performed in the United Kingdom in 1991, examining 70 patients (mean age 62 years) admitted at (a mean of) 4.8 hours after the onset of symptoms; (b) two studies by Pitzalis et al. performed in Italy, published in 1994 [21] and 1998 [22], examining 21 and 59 patients, respectively (mean age of 54 years in both), admitted within six hours; (c) the study by Gonzalez Sanchez et al. [23] performed in Spain in 1998, examining 49 patients (mean age 62 years) admitted within eight hours; (d) the study by Bordalo-Sa et al. [24], performed in Portugal in 1999, examining 45 patients (mean age 56 years) admitted within six hours; and, lastly, e) the study by Doulalas et al. [25] performed in the United Kingdom in 2001, examining 97 patients (less than 70 years of age) admitted at (a mean of ) 5.4 hours after the onset of symptoms. The total number of patients in these studies [20-25], was 341, of whom 165 had anterior and 176 had inferior MI. The quality of all studies [20-25] is satisfactory, as seen in the completed Newcastle-Ottawa Quality Scale assessment (Table 1).

**Table 1:** Newcastle-Ottawa Quality Scale Risk of bias analysis for the included cohort studies [20-25] according to the Newcastle-Ottawa Quality Scale assessment.

	Selection				Comparability		Outcome			Total quality score
Study	Representativeness of exposed cohort	Selection of non-exposed cohort	Ascertainment of exposure	Demonstration that outcome was not present	Adjust for the most important risk factors	Adjust for other risk factors	Assessment of outcome	Follow-up length	Loss to follow-up rate	
Pipilis 1991 [20]	1	1	1	0	1	0	1	1	1	High
Pitzalis 1994 [21]	1	1	1	0	1	0	1	1	1	High
Sanchez 1998 [23]	1	1	1	0	1	0	1	1	1	High
Pitzalis 1998 [22]	1	1	1	0	1	0	1	1	1	High
Bordalo- Sa 1999 [24]	1	1	1	0	1	0	1	1	1	High
Doulalas 2001 [25]	1	1	1	0	1	0	1	1	1	High

The basic characteristics of the six studies [20-25] are shown in Table 2.

**Table 2:** Basic characteristics of the studies entered in meta-analysis.

Study	Pipilis (1991) [20]		Pitzalis (1994) [21]		Sanchez (1998) [23]		Pitzalis (1998) [22]		Bordalo-Sa (1999) [24]		Doulalas (2001) [25]	
Onset of symptoms (hours)	4.8		<6		<8		<6		<6		5.4	
Infarct location	Anterior	Inferior	Anterior	Inferior	Anterior	Inferior	Anterior	Inferior	Anterior	Inferior	Anterior	Inferior
Number of patients	34	36	10	11	22	27	30	29	25	20	50	70
Mean age	62	62	53	55	No data	No data	53	54	56	56	59	59
Gender (male)	28	28	No data	No data	No data	No data	25	23	No data	No data	41	50
Thrombolysis	33	33	No data	No data	No data	No data	29	23	25	20	50	70

Heterogeneity

According to the Q-test, significant heterogeneity was absent (Q=6.30, p=0.27, τ=0.002, I² = 0.0068%), indicative of the estimated average being in the same direction with each individual outcome. No study had a studentized residual greater than 2.63, suggesting absence of outliers, and none could be considered overly influential, according to Cook's distances. Neither the rank correlation (p=0.27) nor the regression test denoted funnel plot asymmetry, although the result of the latter was of borderline significance (p=0.0548). In five studies [20-22,24,25], SDNN was derived from the entire 24-hour strips and in one [23] from five-minute intervals within each recording hour.

Mean differences

**Figure 2:**
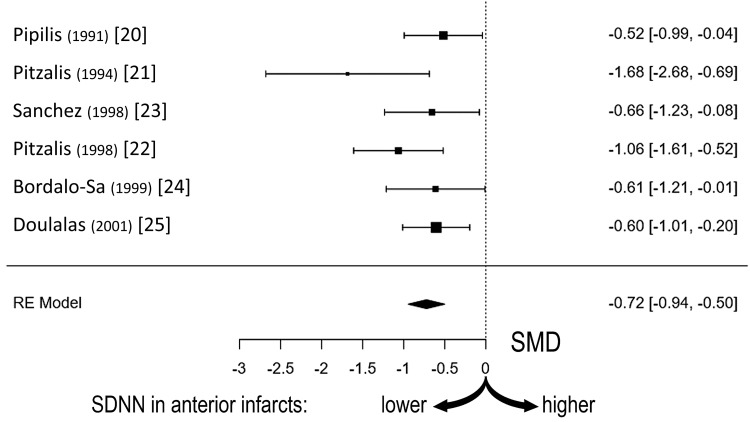
Standardized mean differences Forest plot depicting standardized mean differences (SMD) and confidence intervals of the standard deviation of the interbeat intervals between normal sinus beats (SDNN) of the six studies [20-25]. Values are lower in anterior than in inferior infarcts, indicative of sympathetic and vagal activation, respectively.

The observed standardized mean differences in SDNN ranged from -1.684 to -0.517. As seen in the forest plot (Figure 2), statistical significance was present in all six studies.

**Figure 3:**
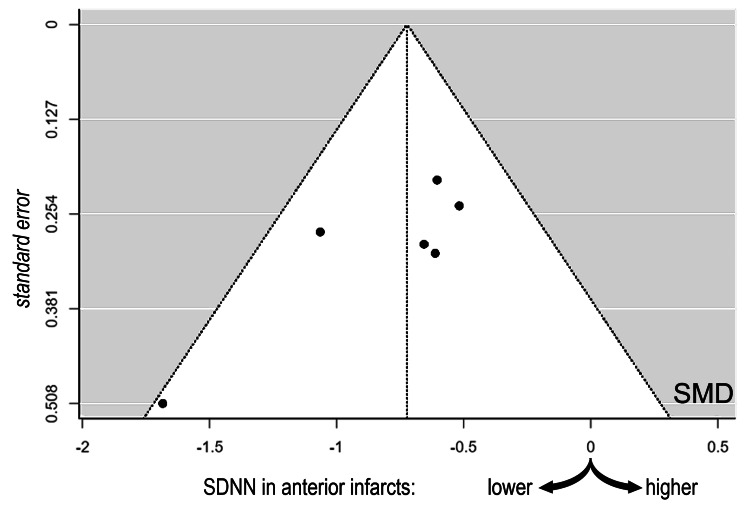
Funnel plot Funnel plot demonstrating the absence of publication bias among the six studies [20-25].

The estimated average of the standardized mean difference (based on the random-effects model) was -0.722 (95% confidence intervals: -0.943 to -0.501), which differed from zero (z=-6.416, p<0.0001). As seen in the funnel plot (Figure 3), publication bias was largely absent, with five studies [20,22-25] plotted near the estimated average and only one [21], displaying relative deviation from this value.

Discussion

Several studies have previously examined autonomic responses post-MI, but only a few focused on the acute phase. Of these, even less reported autonomic indices separately for anterior and inferior MI, a piece of information having pathophysiologic and therapeutic implications. Our present meta-analysis provides further input in this regard, by reporting the widely used index SDNN, which reflects sympathovagal balance [31] and correlates with other HRV indices derived from time- and frequency-domain analysis [32].

HRV during the acute phase

All studies included in our meta-analysis reported consistent differences in SDNN between anterior and inferior MI that were in the same direction as the estimated average. Homogeneity was satisfactory with the absence of outliers, whereas no study could be considered overly influential. Likewise, there was no major funnel plot asymmetry, as demonstrated by the rank correlation and regression tests. However, it should be noted that the power of these tests in distinguishing chance from real asymmetry is relatively low, because of the small number of studies included in the meta-analysis.

We found significant differences in SDNN between anterior and inferior MI, suggesting sympathetic and vagal predominance, respectively. Our result is in keeping with the time-domain analysis in the work of Zabel et al. [14] and Flapan et al. [26] and the frequency-domain analysis in the work of Luria et al. [27]. Taken together, these studies [14,20-27] confirm previous observations [12] and contribute to ongoing discussions regarding early β-blockade in patients with acute MI [33].

Early β-blockade

Treatment with β-adrenergic antagonists improves the outcome after acute MI, but previous reports have raised caution against early initiation, in view of the risk of hemodynamic compromise that offsets antiarrhythmic benefits [34]. Our data indicate that infarct location should be considered in decision-making; thus, treatment may be withheld in patients with inferior MI, a view supported by recent data, demonstrating survival benefits after early β-blockade only in patients with anterior MI [35]. Nonetheless, marked variation exists, underscoring the need for individualized decisions. For instance, HRV analysis in the study by Lombardi et al. [28] failed to demonstrate vagal hyperactivity in inferior AMI, despite the enhanced sympathetic activation in anterior MI; hence, emphasis should be placed on the temporal evolution within the 24-hour timeframe, in the presence or absence of revascularization. To our knowledge, there is only one study [36] examining HRV in the pre-thrombolysis era in 21 patients (11 inferior, 10 anterior MI) during the initial 24 hours; using pNN50, higher vagal activity was reported in patients with inferior MI, a pattern confirmed by attenuation of interbeat variation after atropine administration [36].

Effects of revascularization

Successful revascularization salvages ischemic myocardium at risk of necrosis, thereby improving left ventricular function and overall prognosis. These salutary effects are reflected in the amelioration of autonomic dysfunction during the subacute phase, with responses according to infarct location becoming less discernible. For example, in the study by Zabel et al. [14], reperfusion with thrombolysis enhanced vagal activity mostly in anterior infarcts, a finding associated with a lower incidence of ventricular tachyarrhythmias. The speed of this process appears to vary among revascularization strategies, i.e., primary PCI versus thrombolysis, with delayed improvement in the latter, despite transient, short-lived fluctuations [37]. Another study examined thrombolysis followed by rescue PCI, with HRV measurements performed beyond the acute phase, i.e., (a mean of) 40 hours after the onset of symptoms [38]. In this cohort, the same autonomic pattern was confirmed, with successful thrombolysis resulting in faster HRV recovery in inferior, as compared to anterior MI; of note, rescue PCI exerted minimal effects on HRV indices in either location [38]. 

The effects of primary PCI on HRV were examined in two studies [29,30], published in 2000 and 2015, respectively. The first [29] included 123 consecutive patients, in whom HRV was analyzed (in the time and frequency domains) from 24-hour Holter recordings commenced on admission. Post-PCI, autonomic balance improved irrespective of infarct location, but sympathetic activation remained higher in anterior MI; short-lived HRV fluctuations were present, particularly in early procedures [29]. The second study [30] utilized a novel nonlinear analysis in 33 consecutive patients (15 anterior, 18 inferior); interestingly, reversal of the autonomic pattern was reported (a mean of) 22 hours post-PCI, with vagal and sympathetic dominance in anterior and inferior MI, respectively. These results are intriguing and difficult to interpret, although they require confirmation by implementing various HRV analyses in larger cohorts.

Immediate autonomic responses

The main limitation of our present work lies in the absence of data describing immediate autonomic responses, a crucial component of pre-hospital arrhythmogenesis in patients with acute MI [39]. To overcome the inherent limitations in acquiring early recordings, HRV measurements from recordings under controlled circumstances (such as in patients undergoing PCI) have been proposed. In such a cohort of 73 patients, HRV changes were observed during balloon inflations in approximately one-third, but no distinct pattern was evident in relation to the occlusion site [40]. Specifically, left anterior descending artery occlusion induced vagal and sympathetic activation in 23% and 11% of patients, respectively; circumflex artery occlusion in 26% and 11%; and lastly, right coronary artery occlusion in 26% and 21% [40]. Based on the extremely short duration of ischemia, these findings cannot be extrapolated to the setting of acute coronary syndromes. By contrast, heart rate changes during more prolonged episodes, such as in variant (Prinzmetal’s) angina [41], provide more data regarding the nature of rhythm disturbances immediately following ischemia, resembling the pattern described in patients with acute MI.

The classical work by Pantridge’s group [42] is the capstone of a long-term endeavor that influenced the management of acute MI. It described the relation of autonomic pattern and MI location in 240 patients, monitored within one hour after the onset of symptoms; all were in sinus rhythm and autonomic assessment was made in the absence of confounding factors, such as history of hypertension or treatment with β-blockers. Of the total cohort, 89 patients were seen within 30 minutes, with 83% of them showing evidence of autonomic dysfunction; this incidence fell to 56% among the 151 patients presenting within the second half of the first hour, with vagal overactivity persisting in inferior MI. Overall, the incidence of ventricular fibrillation within one and four hours was 9.5% and 15.5%, respectively [42]. Despite the simplicity of autonomic assessment in this work, its findings underscore the need for further research, focusing on the early hours post-MI. 

Future directions

Primary ventricular tachyarrhythmias have been linked mostly to anterior MI, due to the higher degree of sympathetic activation, the pathophysiologic role of which is well-documented [43]. However, this view has been challenged by the findings of a large meta-analysis, demonstrating comparable incidence between anterior and inferior MI [44]. Thus, the nature and speed of changes in each autonomic arm during acute MI merit specific attention. Preliminary data from our group indicated swift alterations in vagal activity, as opposed to slower sympathetic responses during a 24-hour observation period post-MI in rats [45]. Emphasis should be placed on the differences in afferent stimulation between various ischemic zones, on the interactions between each autonomic arm, and on their effects on the sinus node, the atrioventricular conduction, and the ventricular myocardium [46].

## Conclusions

Our meta-analysis demonstrates sympathetic and vagal dominance during the acute phase of anterior and inferior MI, respectively. Autonomic dysfunction is ameliorated by revascularization, with faster recovery rates observed after PCI (as compared to thrombolysis) and in inferior MI (as compared to anterior MI). Early β-blockade is advisable in anterior MI, whereas it can be deferred in inferior MI, particularly in cases of delayed revascularization. As our meta-analysis totaled only 341 patients, further data focusing on the acute phase is needed that will enable firmer conclusions and treatment recommendations. This applies particularly to the early hours of MI, in view of the associated high incidence and poor prognosis of ventricular tachyarrhythmias. Ongoing research, combining preclinical, clinical, and epidemiologic data, aims at deeper pathophysiologic insights that could lead to effective therapeutic strategies.
